# Unorthodox Transcriptional Mechanisms of Lipid-Sensing Nuclear Receptors in Macrophages: Are We Opening a New Chapter?

**DOI:** 10.3389/fendo.2020.609099

**Published:** 2020-12-10

**Authors:** Zsolt Czimmerer, Laszlo Halasz, Laszlo Nagy

**Affiliations:** ^1^ Department of Biochemistry and Molecular Biology, Faculty of Medicine, University of Debrecen, Debrecen, Hungary; ^2^ Departments of Medicine and Biological Chemistry, Johns Hopkins University School of Medicine, Institute for Fundamental Biomedical Research, Johns Hopkins All Children’s Hospital, St. Petersburg, FL, United States

**Keywords:** macrophage, lipid sensing nuclear receptors, ligand-insensitive role of nuclear receptor, nuclear receptor cistrome, epigenetic regulation

## Abstract

Work over the past 30 years has shown that lipid-activated nuclear receptors form a bridge between metabolism and immunity integrating metabolic and inflammatory signaling in innate immune cells. Ligand-induced direct transcriptional activation and protein-protein interaction-based transrepression were identified as the most common mechanisms of liganded-nuclear receptor-mediated transcriptional regulation. However, the integration of different next-generation sequencing-based methodologies including chromatin immunoprecipitation followed by sequencing and global run-on sequencing allowed to investigate the DNA binding and ligand responsiveness of nuclear receptors at the whole-genome level. Surprisingly, these studies have raised the notion that a major portion of lipid-sensing nuclear receptor cistromes are not necessarily responsive to ligand activation. Although the biological role of the ligand insensitive portion of nuclear receptor cistromes is largely unknown, recent findings indicate that they may play roles in the organization of chromatin structure, in the regulation of transcriptional memory, and the epigenomic modification of responsiveness to other microenvironmental signals in macrophages. In this review, we will provide an overview and discuss recent advances of our understanding of lipid-activated nuclear receptor-mediated non-classical or unorthodox actions in macrophages.

## Introduction

A generally accepted paradigm in endocrinology has been over the last 30 years that if not the sole, but the main function of lipid-sensing nuclear receptors is to translate microenvironmental chemical cues into distinct biological effects through tight regulation of gene transcription. In the 1980s, the cloning of the first nuclear receptors including the glucocorticoid and estrogen receptors was followed by the identification of dozens of evolutionarily related proteins and initiated the development of this concept. Although at the time the newly identified proteins showed similar domain structure containing DNA binding, ligand binding, and transactivation domains, initially the nature of their endogenous chemical ligands was unknown. Therefore, these proteins were called ″orphan″ receptors. The approach termed ″reverse endocrinology″ (having the receptor first and looking for the ligand later) allowed the discovery of dietary lipid-derived, often lower-affinity endogenous ligands, for many of them including liver X receptors (LXRs) and peroxisome proliferator-activated receptors (PPARs) (1, 2). Since the identification of their first endogenous lipid ligands, several natural and synthetic nuclear receptor activity modifier molecules including agonists, antagonists, partial agonists, and inverse agonists have been discovered. This chemical toolbox helped us to learn more about the transcriptional basis of lipid-sensing nuclear receptor actions and the functional consequences of their ligand activation in various cell types including macrophages under different physiological and pathological conditions (3–5). Beyond the classical transcriptional effects, it has been demonstrated that nuclear receptors may have non-genomic activities. Various orphan and lipid sensing nuclear receptors can also be located in the cytoplasm and in the plasma membrane often in lipid rafts. The extranuclear localization of nuclear receptors not only influences transcription through the modulation of the receptor’s availability in the nucleus but also control various biological processes interacting with other signal transduction pathways (6–9). We will not cover these activities in this overview. Importantly, until the end of the 2000s, the applied methods were not particularly suitable to thoroughly investigate the receptors in an unbiased fashion and discover potential nonclassical, but nuclear and chromatin associated functions of lipid sensing nuclear receptors. This is because the widely used approaches were ligand activation centric and biased and researchers were looking for ligand-induced changes with the goal to identify regulated genes. This was a significant bias and severely limited the scope of investigations. The spread of next-generation sequencing-based epigenomic and transcriptomic technologies has changed all this and given new impetus to nuclear receptor research resulting in comprehensive genome-wide maps and led to the identification of a ligand-independent and novel ligand-directed transcriptional regulatory roles of lipid-sensing nuclear receptors. In this review, we will summarize this voyage and our current understanding about nonclassical transcriptional regulatory actions of lipid-activated nuclear receptors including PPARs and LXRs in macrophages. We chose to focus on macrophages due to the remarkable plasticity of this cell type and the relatively large amount of genome-wide and functional analyses available (10–12).

## Transcriptional Basis of Macrophage Heterogeneity and Plasticity

Macrophages play an essential role in the maintenance of normal tissue homeostasis and the protection against different pathogen infections but also participate in different human diseases including atherosclerosis, cancer, and obesity. Phenotypic and functional features of macrophages are tightly determined by the combination of their developmental origin and tissue microenvironment (13, 14). Many tissue-resident macrophage-subsets including brain, liver, lung, and kidney macrophages are derived from fetal progenitors and produce self-renewing populations, while intestinal and dermal macrophages are continuously replenished by bone marrow-derived monocytes. Monocyte-derived macrophages are infiltrating and thus observed at sites of injury and inflammation. Both functional heterogeneity and polarization of tissue-resident and monocyte-derived macrophage subsets are precisely regulated by their complex molecular microenvironment (15, 16). Two extreme endpoints of macrophage polarization are Th1-type cytokine interferon-gamma (IFNγ) or Gram-negative bacteria-derived lipopolysaccharide (LPS)-induced classical [M(IFNγ) or M(LPS)], and Th2-type cytokines interleukine-4 (IL-4) or IL-13-promoted alternative [M(IL-4) or M(IL-13)] polarization. Classically polarized macrophages have inflammatory properties and high antibacterial activity, while alternative macrophage polarization is associated with anti-inflammatory features supporting protection against nematode infections and tissue regeneration. However, numerous transient macrophage polarization states are identified *in vitro* and *in vivo* which can be switched depending on changing microenvironmental milieu (14, 16, 17).

Macrophage identity and response to changing molecular milieu require strict regulation of their gene expression program at the transcriptional level through complex and well-organized collaboration between genomic regulatory regions and DNA-binding transcription factors (TFs) (10, 18). Gene-proximal promoters and distal regulatory elements (so-called enhancers) are associated with characteristic and partially distinct covalent post-translational histone modification patterns and contain several transcription factor-specific DNA motifs. Promoters are marked by H3K4m3, while enhancers exhibit high levels of H3K4m1 and H3K4m2. Besides, both regulatory elements are associated with H3K27Ac following their activation and H3K27m3 in a repressed state (19–21). The available enhancer repertoire is of great importance to specify the identity of the macrophage lineage and is primarily determined by the collaborative binding of general macrophage-specific lineage determining transcription factors (LDTFs) such as PU.1, AP-1, and CEBPβ. The complex interaction between these LDTFs results in chromatin opening, enhancer activation, and new loop formation between promoters and enhancers leading to the formation of macrophage-specific enhancer repertoire (11, 22). Intriguingly, recent studies have raised the possibility that additional transcription factors including GATA6, SALL1, and nuclear receptors can also act as LDTFs participating in the determination of tissue-specific enhancer sets in various tissue-resident macrophages (23–28).

The macrophage subset-specific enhancer repertoires serve as a binding platform for the signal-dependent transcription factors (SDTFs). Many microenvironmental signals including pathogen-derived molecules, cytokines, and lipids can activate SDTFs turning on signal-specific gene expression programs (29–32). Toll-like receptor (TLR) ligands such as LPS and poly(I:C) as well as tumor necrosis factors (TNFs) activate nuclear factor kappa-light-chain-enhancer of activated B cells (NFκB) and Activator protein 1 (AP-1) transcription factor complexes initiating a transcriptional program of the inflammation (33, 34). Various cytokines can activate different members of the signal transducer and activator of transcription (STAT) transcription factor family. Each member of the STAT family binds to different DNA motifs and regulates different gene sets leading to the emergence of distinct macrophage polarization states including IL-4-STAT6 signaling pathway-induced alternative and IFNγ-STAT1 axis-activated classical macrophage polarization (35). Finally, the lipid microenvironment can also directly control the gene expression at the transcriptional level by activation of lipid-sensing nuclear receptors influencing macrophage metabolism and inflammation (3–5). Recently, many *in vitro* and *in vivo* pieces of evidence indicate that different microenvironmental signals can interact with each other at the epigenomic level affecting genome-wide chromatin accessibility, cofactor binding, and enhancer activity in human and murine macrophages. These complex interactions decisively influence transcriptomic profiles of macrophages resulting in complex macrophage phenotypes under different physiological and pathological conditions (36–40). It is the context one needs to consider the role and contribution of nuclear hormone receptors.

## The General Architecture and Regulatory Mechanisms of Nuclear Receptors

The nuclear receptor superfamily contains various transcription factors that act as SDTF and play a crucial role in the signal translation from constantly changing lipid microenvironment to gene expression alterations. This functional complexity is based on the evolutionarily conserved protein structure. All nuclear receptors consist of N-terminal ligand-independent activation function (AF-1), DNA binding (DBD), hinge or linker, ligand binding (LBD), and ligand-dependent terminal activation (AF-2) domains. Highly conserved two zinc-finger motifs containing DBDs are responsible for the recognition and binding of specific DNA sequences known as hormone response elements. More diverse LBDs recognize receptor-specific lipid ligands and form dimerization surfaces, while AF-2 domains within LBDs create a binding surface for coactivator and corepressor complexes (41).

The nuclear receptor superfamily includes both classical endocrine receptors such as receptors for steroid hormones, thyroid hormones, and fat-soluble vitamin A or D and various orphan receptors whose ligands were initially unknown. Since their discovery, many orphan receptors become ″adopted″ by identification of their specific endogenous ligands for instance oxysterols for LXRs or short-chain fatty acids for PPARs. However, several receptors remained orphans without known endogenous ligands (42). Despite structural similarities, ligand-sensitive nuclear receptors show significant differences in their mechanisms of action. The first type of these receptors includes steroid receptors and can be found in the cytoplasm associating with heat-shock proteins in an unliganded state. Ligand activation of steroid receptors leads to the dissociation from heat-shock proteins, homo-dimerization, translocation into the nucleus, and binding their specific hormone-responsive DNA elements activating their target genes at the transcriptional level. The second type of ligand-sensing nuclear receptors including various classical hormone and dietary lipid-sensing receptors form heterodimers with RXRs and bind constitutively to DNA in the nucleus regardless of their ligand binding states, but their interaction partners and functional properties are tightly dependent on the presence of their ligands (43). In an unliganded state, these heterodimers interact with corepressor proteins such as silencing mediator of retinoic acid and thyroid hormone receptor (SMRT) and nuclear receptor corepressor (NCoR) complexes and attenuate basal mRNA expression levels of their target genes. Ligand binding induces conformation changes in the LBD leading to corepressor/coactivator exchange and consequential transcriptional activation (44). On the other hand, several liganded nuclear receptors can also inhibit the transcription activator activity of another SDTFs through transrepression. This transcriptional repressor mechanism is based on protein-protein interactions without direct sequence-specific DNA binding of nuclear receptors and associated with sumoylation and corepressor complex recruitment (4, 45).

## The Classical Role of Dietary Lipid-Sensing Nuclear Receptors in the Regulation of Macrophage Metabolism and Inflammation

Several lipid-sensing nuclear receptors including LXRs, PPARγ, PPARδ, and their heterodimerization partners RXRα and β are expressed in macrophages and their expression levels and ligand-dependent activities are tightly regulated by various microenvironmental signals (46–48). The classical paradigm of nuclear receptor biology relies on lipid-sensing nuclear receptors to form a bridge between macrophage metabolism and inflammation (3, 43). This notion was supported by several lines of evidence: i) endogenous ligands for PPARs and LXRs are small lipid molecules; ii) macrophages are often present in a lipid-rich environment and are themselves metabolically active cells; iii) metabolic and inflammatory genes are regulated in parallel but with different mechanisms by endogenous or synthetic agonists-activated lipid-sensing nuclear receptors. A good example of the metabolic role of lipid-sensing nuclear receptors is that liganded PPARγ and LXRs tightly control cholesterol transport and storage in macrophage-derived foam cells from atherosclerotic lesions. oxLDL induces PPARγ expression and oxLDL-derived 9-HODE and 13-HODE serve as endogenous ligands for PPARγ. Liganded PPARγ enhances further oxLDL uptake through the increase of the scavenger receptor CD36 expression, the LXR, and the endogenous LXR ligand 27-hydroxicholesterol producing enzyme CYP27A1 expressions. LXR activation leads to elevated cholesterol efflux through induction of ABCA1 and ABCG1, facilitated intracellular cholesterol trafficking by enhancement of NPC1 and 2 expressions, as well as attenuated cholesterol uptake. The latter process is mediated by liganded LXR-induced E3 ubiquitin ligase inducible degrader of the LDLR (IDOL), which triggers proteasomal degradation of LDLR and VLDLR [reviewed in (49)]. In addition to their metabolic roles, lipid sensing nuclear receptors modify the immunological features of the macrophages. On the one hand, both ligand-activated PPARγ and LXRs can repress various inflammatory signal-activated transcriptional programs in macrophages through inhibition of different SDTFs such as IFNγ-activated STAT1 or LPS-activated NFκB and AP-1 transcription factor complexes [reviewed in (4, 45)]. On the other hand, liganded LXRs also promote phagocytic capacity and survival in macrophages, while PPARγ controls the regenerative activity of muscle infiltrating macrophages following muscle injury (50–52).

Importantly, however, LXRs and PPARs act in permissive heterodimers with RXRs meaning that these heterodimers can also be activated by ligands of both RXRs and LXRs or PPARs (53). Initially, 9-cis-retinoic acid was a widely accepted endogenous RXR ligand, but it proved to be difficult to detectable under physiological conditions in vertebrates, thus raising doubts about its *in vivo* relevance. However, several pieces of evidence show that 9-cis-13,14-dihydro retinoic acid meets better the criterion of a physiological RXR ligand (54, 55). Even though the true identity of the endogenous RXR ligand(s) is one of the remaining mysteries of nuclear receptor biology, various studies demonstrated that synthetic RXR ligands can activate an RXR-specific transcriptional program in macrophages resulting in the changes of their phenotypic and functional characteristics (56, 57). It has been shown that RXR ligand activation leads to elevated VEGFα production, enhanced leukocyte migration, and altered inflammatory response and metabolism (57, 58). The majority of liganded RXR-regulated genes are overlapping with LXR or PPAR-activated gene signatures, but some experimental evidence shows that RXR can also act as a homodimer or in a heterodimer with orphan nuclear receptors such as Nur77 (57–60). These findings indicate that RXR ligand activation results in a unique transcriptional and biological responses in macrophages thus RXR is more than a “simple and silent” interaction partner for PPARs and LXRs.

## Nuclear Receptor Ligand Sensitivity From an Evolutionary Perspective

If one wants to study the ligand responsiveness of nuclear receptors from a broad perspective, it is useful to take an evolutionary point of view. The ancient and conserved nuclear receptor superfamily believed to emerge in the metazoan lineage, but significant differences can be observed in the number of encoded nuclear receptors between different species. Notably, 2 nuclear receptors have been identified in the sponge *Amphimedon queenslandica*, 284 in the *Caenorhabditis elegans* (*C. elegans*), 21 in the fruit fly, 33 in the amphioxus, 47 in the rat, 49 in the mouse, and 48 in the human genome (61–65). According to the currently accepted view, nuclear receptors originate from ancestral fatty acid sensors of sponges, and the evolutionary shifts in ligand preference are the consequences of mutations altering the ligand-binding cavity (61). Furthermore, the evolution of ligand binding is not simple ligand-receptor coevolution, because the nuclear receptor ligands are not proteins but products or intermediates of various metabolic pathways such as isoprenoids, fatty acids, or fatty acid metabolites. Consequently, certain nuclear receptors may be activated by completely different ligands during an early evolution compared to the mammals (66). However, approximately half of the nuclear receptors in mammals fall into subclass lacking traditional ligands and the proportion of ligand-responsive nuclear receptors is very low in many primitive invertebrate species. For instance, one out of 284 nuclear receptors have been identified as ligand-responsive in the *C. elegans*, while only two out of 21 nuclear receptors have traditional ligands in the fruit fly (65, 67). These findings indicated that the nuclear receptor/ligand evolutionary relationship is very complex and dynamic, but ligand-independent transcriptional regulatory activities of nuclear receptors are important from primitive invertebrates to humans. Nevertheless, until recently a potential ligand insensitive action of metabolite sensing nuclear receptors was difficult to investigate.

## Next-Generation Sequencing-Based Methodologies as a Transforming Tool for Discovery of New Layers in Nuclear Receptor-Mediated Transcriptional and Epigenomic Regulation

**Figure 1:**
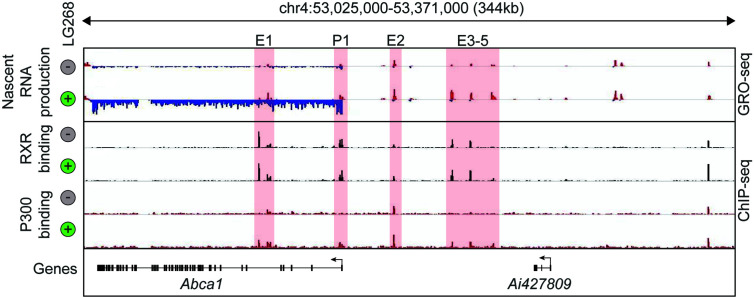
Definition of putative RXR regulated enhancers. Genome browser view on the ABCA1 locus. GRO-seq and ChIP-seq results for RXR and P300 are shown in control (Veh, 60 min) and stimulated (LG268, 60 min) macrophages. Putative enhancers are highlighted (E1-E5 and P1).

In the last decade, the development and expansion of next-generation sequencing-based methodologies in transcriptomics and epigenomics contributed to the better understanding of the transcriptional basis of cell specification and cellular responses to the changing microenvironment. Among these methods, Chromatin Immunoprecipitation Sequencing (ChIP-seq) is routinely used to study the genome-wide binding of transcription factors and cofactors as well as post-translational histone modification patterns in eukaryotic cells. Assay for Transposase Accessible Chromatin Sequencing (ATAC-seq) is suitable for the identification of open chromatin regions, while Global Run-On Sequencing (GRO-seq) can detect and quantify the nascent RNA expression (68–70). Therefore, key questions such as: Where does chromatin open? Where does a particular transcription factor bind? and Where is transcription initiated? can be answered by covering the entire genome in an unbiased manner. The combination of RNA sequencing-based global transcriptome analysis with these techniques helped to identify new layers of connection between the genome-wide binding of LDTFs and SDTFs (i.e. their cistromes), cell state-specific active enhancer landscape, and their transcriptional output. Thereby, it became possible to carry out the systematic analysis of nuclear receptor binding and function in different cellular systems. The initial ChIP-seq studies confirmed many elements of our prior knowledge about non-steroid nuclear hormone receptors including nuclear localization and DNA and chromatin binding in the unliganded state or their binding to receptor-specific hormone response elements but also resulted in some unexpected findings. For instance, a single nuclear receptor can bind numerous (10.000–25.000) sites in the genome and a not negligible part of the nuclear receptor-bound regions does not contain known hormone response elements (1). Comparing the number of nuclear receptor-bound genomic regions to ligand-activated genes, it could be observed that a large number of nuclear receptor-bound genomic sites are associated with a relatively small number (250–1.000) of ligand-responsive genes. It was also demonstrated that genome-wide nuclear receptor binding may be significantly rearranged after molecular microenvironmental changes (1, 71, 72). Besides, the combination of nuclear receptor-specific ChIP-seq with quantification of nascent RNA expression at genomic regulatory and coding regions by GRO-seq allowed the investigation of the ligand-dependent direct transcriptional regulatory role of nuclear receptors. The liganded nuclear receptor-regulated enhancers could be identified in different cell types using the following simple, correlative criteria: i) nuclear receptor binding; and ii) dynamically changing nuclear receptor ligand-induced or repressed nascent RNA expression at the given regulatory regions. These sites then can be annotated to the closest similarly regulated gene. A representative example is shown in **Figure 1** and (57). Interestingly, these studies also revealed that a portion of nuclear receptor-bound enhancers is insensitive to ligand stimulation indicating a potential ligand-independent function (73–76). Overall, these early studies suggested that cell-type and cellular state-specific nuclear receptor cistromes are tightly dependent on other LDTFs or SDTFs and lipid-sensing nuclear receptors may also act in a non-classical, ligand-independent way.

## Lipid Sensing Nuclear Receptors in the Specification of Tissue-Resident Macrophage Subsets and as Modulators of Inflammatory Response

**Figure 2:**
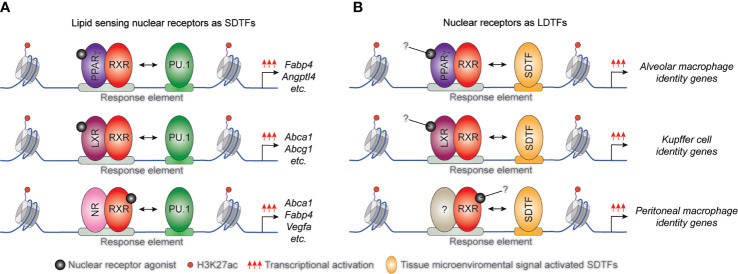
The position of macrophage-expressed lipid sensing nuclear receptors in transcription factor hierarchy shows gene and tissue-dependency. **(A)** General macrophage lineage-specific LDTFs such as PU.1 contribute to the classical STDF action and ligand-mediated transcriptional activation capacity of lipid sensing nuclear receptors controlling macrophage metabolism. **(B)** Lipid sensing nuclear receptors also can act as LDTFs determining the tissue-specific characteristics of various tissue-resident macrophage subsets.

Several studies demonstrated that the interactions between lipid-sensing nuclear receptors and LDTFs are largely macrophage subset and microenvironment-specific. It has been described that the macrophage-specific LDTF PU.1 plays a central role in the formation of macrophage-specific cistromes for various SDTFs and is required for SDTF-directed transcriptional programs in murine BMDMs and macrophage cell line. Both the LXRα cistrome and LXR agonist GW3965-induced expression of various selected LXR target genes including *Elovl5*, *Abca1*, and *Abcg1* was diminished in the absence of PU.1. Conversely, PU.1 binding, and the active enhancer mark H3K4m1 pattern did not show any differences in LXRα/β double deficient BMDMs (**Figure 2A**) and (22). Applying combined bioinformatic and ChIP-seq approaches, we reported that PPARγ binding also depends on both PU.1 binding and quality of PPARγ-specific DR1 motif in macrophages (77). However, the systematic analysis of murine tissue-resident macrophage enhancer landscapes identified many nuclear receptor-binding motifs in the macrophage subset-specific enhancer clusters including LXRα binding motif in the Kupffer cells and splenic macrophages or PPARγ binding DNA element in the splenic and alveolar macrophages. These findings raised the possibility that these lipid-sensing nuclear receptors may also act as LDTFs in different tissue-resident macrophages (78). In recent years, this hypothesis has been confirmed by several independent studies. Sakai and colleagues described the crucial role of liver-specific molecular microenvironment including hepatocytes-derived LXR ligand desmosterol and sinusoidal endothelial cell-produced Notch ligand DLL4 and TGFβ in the initiation and maintenance of Kupffer cell identity in murine diphtheria toxin-induced Kupffer cell ablation model. DLL4 rapidly induces LXRα expression in repopulating monocytes and LXRα acts as LDTF in collaborative interactions with TGFβ and Notch signaling pathways during Kupffer cell differentiation (28). It has also been observed that diet-induced non-alcoholic steatohepatitis induces changes in the expression levels of collaborative LDTFs including downregulation of SPI-C and upregulation of ATF3 leading to altered binding and function of LXRs in Kupffer cells. The rearranged LXR cistrome contributes to disease-specific gene expression patterns and phenotype in this macrophage subtype (79). The crucial role of LXRα was identified in the differentiation of macrophages in the marginal zone of the spleen by A-Gonzales and colleagues. It was found that marginal zone macrophage specification is defective in LXRα-deficient mice resulting in abnormal responses to blood-borne antigens. The lack of marginal zone macrophages was restored in LXRα-deficient mice by myeloid-specific expression of LXRα or the adoptive transfer of wild-type monocytes (27). The lineage-determining role of PPARγ was also confirmed in alveolar macrophages in *in vivo* mouse experiments. Schneider et al. demonstrated that GM-CSF induces PPARγ expression in fetal monocytes which is responsible for the determination of the perinatal differentiation and the identity of alveolar macrophages through the regulation of several transcription factors and the alveolar macrophage differentiation and function-linked genes (26). It has been previously described that retinoic acid receptor (RAR) activation is required for the functional specialization of peritoneal macrophages through direct induction of GATA6 transcription factor expression (23). However, it has recently been reported that the RXRs themselves can also contribute to the neonatal expansion of large peritoneal macrophage pool and survival of adult large peritoneal macrophages through the regulation of chromatin accessibility and peritoneal macrophage-specific gene signature (80). In addition, Fonseca and colleagues recently showed that PPARγ is an essential collaborating factor for an AP-1 transcription factor complex in resting murine thioglycollate elicited macrophages. On the one hand, the AP1 transcription factor complex member, Jun binding was markedly reduced in PPARγ deficient macrophages at a specific enhancer set, while ATF3 and JunD bindings were not affected. On the other hand, complex protein-protein interactions were observed between PPARγ and AP-1 family members including Jun, JunD, and ATF3. Nevertheless, the functional consequences of the collaboration between PPARγ and AP-1 transcription factor complex are still not completely understood (81).

Genome-wide epigenomic approaches also allowed the testing of the proposed transrepression mode of anti-inflammatory gene regulation by nuclear hormone receptors, which effect inflammatory as well as tissue specific macrophages. The pre-genomic models of lipid sensing nuclear receptor-mediated anti-inflammatory actions were mainly based on the sumoylation-dependent transrepression of various inflammatory signals-activated TFs or TF complexes including NFkB, AP-1 and STAT1 (3, 4, 45) In agreement with the previous studies, the application of genomic approaches could confirm some elements of this regulatory mechanism including the necessity of SMRT and NCoR corepressor proteins for LXR-mediated transrepression (82). However, many questions about transrepression are still waiting for an answer at the whole-genome level. Perhaps the most important of these is the details of the overlap, extent and specificity of the interactions between liganded nuclear receptors and the inflammatory signals-activated transcription factors. In addition, recent studies identified additional mechanisms of liganded lipid sensing nuclear receptor-dependent inhibition of inflammation. Thomas and colleagues showed that liganded LXR can bind directly to inflammatory gene enhancers containing LXR binding sites independently from AP-1 transcriptional factor complex leading to reduced chromatin openness and inflammatory responsiveness (83). Interestingly, it has been also demonstrated that LXR can inhibit inflammatory gene expression through the ligand-dependent induction of Abca1-mediated cholesterol efflux and membrane lipid reorganization rather than transrepression (83, 84). Thus the transrepression mechanism is not fully validated yet and requires additional studies.

These findings indicate that lipid sensing nuclear receptors can also act as macrophage subtype-specific LDTFs (**Figure 2B**) and having a much broader impact on macrophage biology, including the inflammatory response than previously thought and raising the issue that these activities might not be all requiring ligand activation.

## Lipid-Sensing Nuclear Receptor-Directed Ligand Insensitive Regulatory Mechanisms in Macrophages

**Figure 3:**
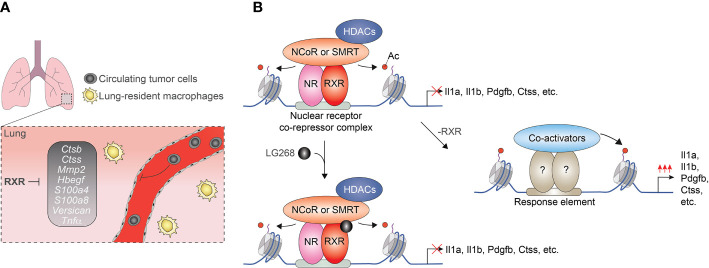
Myeloid-cells-expressed RXRs suppress lung metastases formation in a ligand-independent manner. **(A)** RXRs repress the pro-metastatic gen set in murine lung-derived myeloid cells. **(B)** RXRs interact with SMRT and NCoR corepressor complexes and act as a direct transcriptional repressor regardless of the presence of RXR agonists.

After the initial observations indicating ligand insensitive fraction of nuclear receptor cistromes, several genome-wide studies investigated the ligand responsiveness of lipid sensing nuclear receptors in macrophages. Interestingly but not unexpectedly, our ChIP-seq and GRO-seq-based analysis demonstrated that only 13% of the identified RXR peaks (718/5206) are associated with significantly regulated nascent RNA expression following RXR agonist LG268 treatment in non-polarized murine BMDMs. In the case of the remaining part of the RXR cistrome, RXR binding is observed at transcriptionally silent (GRO-seq negative) or transcriptionally active (GRO-seq positive) but LG268 insensitive genomic regions. These findings suggest that a significant part of RXR cistrome is ligand insensitive or just responds to ligands of heterodimerization partners (57). Although the biological significance of ligand insensitive RXR cistrome is not completely understood in macrophages, our recent study demonstrated that it can play important roles in the suppression of a metastasis-promoting transcriptional program. We observed that myeloid-specific RXR deficiency leads to enhanced lung metastasis formation without influencing primary tumor growth in murine Lewis lung carcinoma (LLC) and B16-F10 melanoma tumor models. This prometastatic phenotype of RXR deficient myeloid cells is characterized by the elevated expression of prometastatic gene signature as well as increased cancer cell migration and invasion promoting capacity (**Figure 3A**). The repressive activity of RXR is based on direct DNA binding of the receptor together with silencing mediator of retinoic acid and thyroid hormone receptor (SMRT) and nuclear receptor corepressor (NCoR) corepressors and is largely insensitive to RXR ligand activation (**Figure 3B**) (85). Recently, the synthetic agonist- and antagonist-insensitive (so-called pharmacologically non-responsive) fractions of LXRα and β cistromes were also identified in non-polarized murine immortalized BMDMs further confirming those opinions that the ligand insensitive fraction of lipid sensing nuclear receptor cistromes is general rather than cell type or nuclear receptor-specific phenomenon (86).

**Figure 4:**
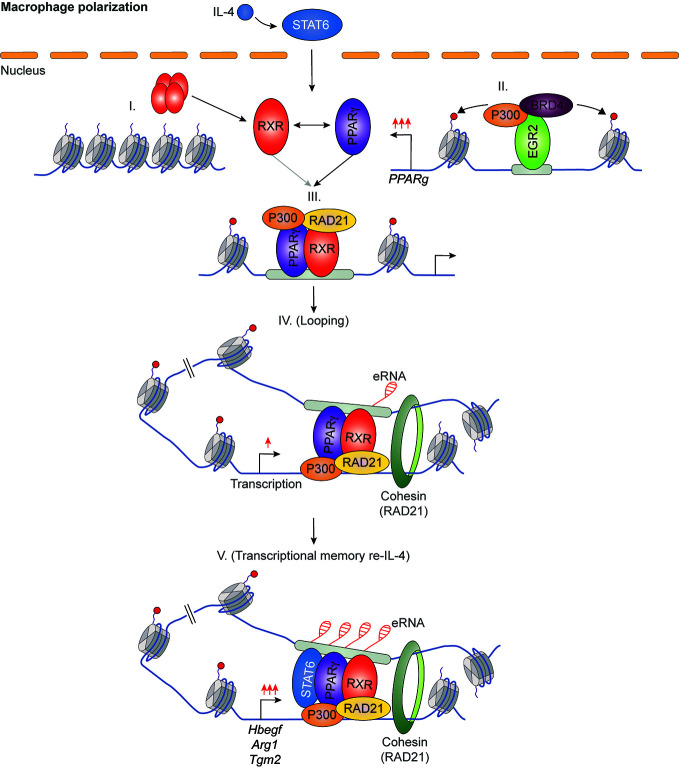
IL-4/STAT6/EGR2 axis-induced PPARγ acts as an epigenomic ratchet in a ligand-independent manner in alternatively polarized macrophages resulting in transcriptional memory and enhanced gene-specific responsiveness to IL-4 re-stimulation.

**Figure 5:**
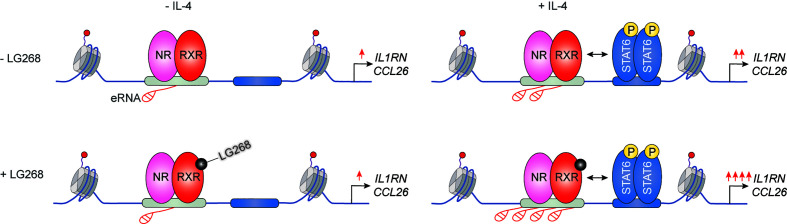
Synthetic RXR agonist supports a gene-specific elevated IL-4 response without any effect on basal gene expression in human differentiating macrophages.

It has been previously described that IL-4 can enhance the ligand-dependent activity of PPARγ in human and murine alternatively polarized macrophages through three different mechanisms including EGR2 transcription factor-dependent activation of its expression, induction of endogenous ligand producing mechanisms, and direct protein-protein interaction with IL-4-activated STAT6 (87–90). Nevertheless, a significant contradiction can be observed between the PPARγ-dependency of alternative macrophage polarization and PPARγ ligand-activated gene expression signature. Odegaard and colleagues demonstrated that PPARγ is necessary for proper alternative macrophage polarization. PPARγ deficiency in myeloid cells impairs alternative macrophage polarization in mice predisposing the animals to the development of diet-induced obesity, insulin resistance, and glucose intolerance (91). However, alternatively polarized human and murine macrophages have PPARγ ligand responsiveness and can produce endogenous ligands, but PPARγ ligand activation cannot induce alternative polarization-linked genes in IL-4-exposed human and murine macrophages (88). Overall, these contradictory findings raised the possibility that PPARγ controls alternative macrophage polarization in an unorthodox and potentially ligand-independent manner. To solve this mystery, we systematically investigated genome-wide RXR and PPARγ bindings and evaluated the PPARγ/RXR heterodimer-directed transcriptional events in the presence and absence of their specific ligands in alternatively polarized murine BMDMs using the combination of next-generation sequencing-based approaches including ChIP-seq, GRO-seq, and ATAC-seq. We observed that both RXR and PPARγ cistromes are expanded in the applied long- and short-term alternative macrophage polarization models following 6-day or 24-h IL-4 stimulation. Interestingly, the IL-4-expanded RXR cistrome is not associated with either IL-4-induced RXR expression or IL-4-reduced RXR binding at many genomic sites further confirming our theory about the existence of a non-chromatin associated RXR pool in the nucleus (92). The expansion of genome-wide RXR and PPARγ bindings is directed by STAT6. The newly formed PPARγ/RXR co-peaks are associated with IL-4-induced chromatin accessibility, PU.1, P300, and RAD21 bindings (56, 93). Although we could identify ligand-activated and repressed PPARγ/RXR heterodimer-bound enhancers with GRO-seq and RNAPII-specific ChIP-seq methods, the majority of PPARγ cistrome were insensitive to both nuclear receptor ligands and IL-4 (56, 93). The ligand insensitive PPARγ cistrome is associated with IL-4-induced and PPARγ-dependent chromatin accessibility as well as P300 and RAD21 bindings. These genomic regulatory elements are responsible for facilitated STAT6 signaling and induction of extracellular matrix-related gene set after second IL-4 stimulation, indicating that ligand insensitive PPARγ acts as an epigenomic ratchet and provides transcriptional memory in alternatively polarized macrophages (**Figure 4**) (93). We also studied the IL-4-induced rearrangement of genome-wide RXR binding in human CD14+ monocyte-derived, differentiating macrophages. Unlike murine BMDM-based long- and short-term alternative macrophage polarization models, RXR cistrome is not expanded after very short (30 min) IL-4 stimulation, but it shows extensive overlap with IL-4-activated STAT6 cistrome in this experimental model. Examining a limited number of IL-4-activated genes and their RXR/STAT6 co-bound enhancers, we could distinguish three distinct interaction types between RXR and IL-4-STAT6 signaling pathways based on the modulatory effect of RXR agonist LG268 on basal gene expression and IL-4 responsiveness: i) basal and IL-4-dependent gene expression and enhancer activations are insensitive for liganded RXR; ii) RXR agonist activates transcription alone and acts synergistically with IL-4; iii) RXR agonist enhances IL-4-dependent transcriptional activations without influencing basal gene expression. The latter suggests a novel function of liganded RXR that it potentiates the macrophage response to other microenvironmental signals without affecting basal gene expression in a gene-specific manner (**Figure 5**) (94). Overall, these findings show that lipid-sensing nuclear receptors play a multifaceted role in macrophages through classical ligand-dependent and novel ligand-insensitive transcriptional regulatory activities.

## The Potential Determinants of Ligand Sensitivity of Lipid-Sensing Nuclear Receptors: From the Facts to the Theories

Even though the above-mentioned studies indicate that a part of lipid-sensing nuclear receptor cistromes is ligand insensitive, the factors affecting their enhancer-specific ligand sensitivity are partially enigmatic. Our knowledge about the three-dimensional structure of lipid sensing nuclear receptors is essential to solving this enigma. In the last two decades, the structural biologists extensively studied the three-dimensional structures of lipid-sensing nuclear receptor complexes such as PPARγ:RXR and LXR : RXR heterodimers contributing to the better understanding of their interactions between various ligands, co-factors, or DNA (95–98). Initially, we thought that the ligand-dependent activation of nuclear receptors is an obvious ‘on-off’ switch, but the structural studies modified this theory. For example, recent findings show that different synthetic PPARγ agonists can interact with both a known ligand-binding pocket of LBD and an alternate binding surface resulting in the complex output of PPARγ activation in the presence of endogenous ligands or synthetic agonists (99). Moreover, endogenous metabolites such as serotonin derivates and butyrate also bind to non-canonical ligand-binding surfaces of PPARγ leading to its activation (100, 101). Overall, these results suggest that lipid-sensing nuclear receptors can integrate different signals from distinct signaling pathways. Therefore, the activation of the pharmacologically insensitive portion of lipid sensing nuclear receptor cistromes *via* the binding of endogenous metabolites to non-canonical ligand-binding surfaces cannot be completely excluded.

It has been also demonstrated that the nucleotide sequence of the binding motif can modulate the three-dimensional nuclear receptor structures and their interactions with DNA influencing the ligand sensitivity. Studying the PPARγ cistrome in alternatively polarized murine BMDMs, we found that the extended DR1 motif was significantly enriched at the synthetic agonist rosiglitazone-activated enhancers. In contrast, ligand insensitive regulatory regions lack this extension and harbor a shorter, more canonical RXR binding site (93). It has been shown that the identified extra 5’ sequence (A-G/C-T) in DR1 can affect the DNA binding affinity of PPARγ:RXR heterodimer and is essential for the PPARγ hinge region to form an interaction with DNA. This interaction is required for the proper conformation and the ligand-binding ability of the receptor suggesting that the PPARγ:RXR heterodimer conformation is suboptimal for the binding of the ligand in the absence of DR1 extension in ligand insensitive enhancers (93, 95, 102, 103). Both pharmacologically sensitive and insensitive parts of LXR cistromes were associated with LXR-specific DNA elements similar to PPARγ, but the extension of LXR-response elements was not identified at the pharmacologically sensitive genomic sites (86). Taken together, these results indicate that the sequence of nuclear receptor binding motifs is one determinant of ligand responsiveness, but not the only one.

The lipid sensing nuclear receptor conformation and activity are also regulated in a ligand-independent manner by covalent post-translation modifications. These include acetylation, phosphorylation, O-GlcNacylation, SUMOylation, or ubiquitination at numerous modification sites influencing different features of nuclear receptors including ligand sensitivity and trans-activation capacity [reviewed in (104, 105)]. Many of them generally affect the activity of nuclear receptor signaling pathways in various cell types, but some modifications can influence the expression of a specific subset of nuclear receptor target genes. For instance, PPARγ Ser273 phosphorylation does not affect the adipogenic capacity of PPARγ but attenuates PPARγ ligand-induced activation of a specific subset of target genes promoting insulin sensitivity *via* inhibited recruitment of Thyroid hormone receptor-associated protein 3 (THRAP3) (76, 106). It has also been described that LXRα phosphorylation at Ser196 regulates its target gene selectivity in macrophages. Chemical inhibition of Ser196 phosphorylation and generation of LXRα S198A phosphorylation-deficient mutant leads to the identification of specific changes in LXR/RXR regulated gene expression. Some LXR target genes such as AIM and LPL showed significantly enhanced LXR agonist-dependent induction in LXRα S198A phosphorylation-deficient mutant but others including ABCA1 or SREBPc1 proved to be insensitive to phosphorylation. Interestingly, the S198A mutation or chemical inhibition of phosphorylation also resulted in significantly elevated basal and LXR ligand-induced CCR7 and CCL24 expression levels (107, 108). These findings indicate that the post-translational modifications of nuclear receptors can attenuate gene-specific responsiveness to various endogenous and synthetic nuclear receptor ligands but their contribution to ligand insensitive nuclear receptor cistromes is currently unknown.

## Concluding Remarks

We have attempted to illustrate above that the transcriptional regulatory role of lipid sensing nuclear receptors is much more comprehensive in macrophages than previously suspected. Although previously we and others have identified many lipid sensing nuclear receptor-activated pathways, our knowledge was limited to the regulation of macrophage metabolism and inflammation through ligand-induced direct transcriptional activation and transrepression. This was because our studies and methods were biased toward ligand-regulated events. Recent progress in epigenomic and transcriptomic methodologies has greatly increased our understanding of different aspects of nuclear receptor biology including their relationships with other LDTFs and SDTFs or their non-canonical transcriptional regulatory actions. Using these approaches, both lineage-determining and ligand insensitive activities of lipid sensing nuclear receptors were identified. These novel transcriptional regulatory mechanisms contribute to tissue-resident macrophage subtype specification, organization of chromatin structure, regulation of transcriptional memory, and modification of responsiveness to other microenvironmental signals. A systematic investigation of the molecular background of newly identified regulatory functions will be necessary for re-thinking of the importance of lipid sensing nuclear receptors in macrophage biology. However, this will take a considerable amount of time and the integration of methodologies of various disciplines including structural biology and epigenomics with genome editing technologies. Furthermore, the application of *in vivo* chemical affinity capture and massively parallel DNA sequencing (Chem-seq) is suitable method to identify the genomic sites bound by small chemical molecules including nuclear receptor activity modifier molecules (109, 110). Therefore, its combination with the nuclear receptor-specific ChIP-seq and GRO-seq methods may help to determine whether the ligand insensitive portion of nuclear receptor cistromes can bind ligand without transcriptional response or is unable to ligand binding. Finally, additional immunological approaches would need to analyze the *in vivo* functional consequences of ligand-independent actions of lipid sensing nuclear receptors in macrophages under different physiological and pathological conditions. After all, we will be able to assess whether these transcriptional regulatory mechanisms play a significant role in the development and progression of various human immunological diseases. Also, there is no reason to believe that nuclear receptors in other cell types are not behaving the same way as in macrophages. These studies requiring comprehensive and unbiased analyses should keep us as a research field occupied for the foreseeable future.

## Author Contributions

All authors listed have made a substantial, direct and intellectual contribution to the work and approved it for publication.

## Funding

Support for this work in the Nuclear Receptor Research Laboratory was provided by the National Research, Development, and Innovation Office KKP129909, K124298, and FK132185 and the European Union and the European Regional Development Fund [GINOP-2.3 2–15-2016-0006]. LN was supported by the National Institutes of Health (R01DK115924). ZC was supported by the Premium Postdoctoral Fellowship Program of the Hungarian Academy of Sciences.

## Conflict of Interest

The authors declare that the research was conducted in the absence of any commercial or financial relationships that could be construed as a potential conflict of interest.
